# Gene Expression Profiling of a Mouse Model of Pancreatic Islet Dysmorphogenesis

**DOI:** 10.1371/journal.pone.0001611

**Published:** 2008-02-20

**Authors:** Laura Wilding Crawford, Elizabeth Tweedie Ables, Young Ah Oh, Braden Boone, Shawn Levy, Maureen Gannon

**Affiliations:** 1 Department of Medicine, Division of Diabetes, Endocrinology, and Metabolism, Vanderbilt University Medical Center, Nashville, Tennessee, United States of America; 2 Department of Molecular Physiology and Biophysics, Vanderbilt University Medical Center, Nashville, Tennessee, United States of America; Children's Hospital Boston, United States of America

## Abstract

**Background:**

In the past decade, several transcription factors critical for pancreas organogenesis have been identified. Despite this success, many of the factors necessary for proper islet morphogenesis and function remain uncharacterized. Previous studies have shown that transgenic over-expression of the transcription factor Hnf6 specifically in the pancreatic endocrine cell lineage resulted in disruptions in islet morphogenesis, including dysfunctional endocrine cell sorting, increased individual islet size, increased number of peripheral endocrine cell types, and failure of islets to migrate away from the ductal epithelium. The mechanisms whereby maintained Hnf6 causes defects in islet morphogenesis have yet to be elucidated.

**Methodology/Principal Findings:**

We exploited the dysmorphic islets in Hnf6 transgenic animals as a tool to identify factors important for islet morphogenesis. Genome-wide microarray analysis was used to identify differences in the gene expression profiles of late gestation and early postnatal total pancreas tissue from wild type and Hnf6 transgenic animals. Here we report the identification of genes with an altered expression in Hnf6 transgenic animals and highlight factors with potential importance in islet morphogenesis. Importantly, gene products involved in cell adhesion, cell migration, ECM remodeling and proliferation were found to be altered in Hnf6 transgenic pancreata, revealing specific candidates that can now be analyzed directly for their role in these processes during islet development.

**Conclusions/Significance:**

This study provides a unique dataset that can act as a starting point for other investigators to explore the role of the identified genes in pancreatogenesis, islet morphogenesis and mature β cell function.

## Introduction

Despite the recent success with islet transplantation as a treatment for replacing insulin-producing β cells lost in individuals with Type 1 diabetes [1], the relative shortage of donor tissue necessitates the development of *in vitro* systems to grow functional islets. Studies by various laboratories over the past several years have resulted in the identification of several transcription factors that function in normal pancreatic/islet cell development (reviewed in [2]); however, much less is known about the cell surface or extracellular components involved in islet formation and function. Ultimately, the generation of optimally functioning islets *in vitro* will likely rely on a complete understanding of how transcription factor networks and cell-cell interactions regulate proliferation, differentiation, and morphogenesis of normal pancreatic endocrine cells.

During pancreas development, islets are formed through a series of morphogenetic events involving cell migration, cell sorting, and cell adhesion. Similar to *Drosophila* and mammalian neurogenesis, pancreatic endocrine cells, of which islets are composed, become committed to the endocrine cell lineage via the Notch signaling pathway [3], [4], [5], [6] and delaminate from the ductal epithelium. In the mouse, the endocrine cells that will go on to contribute to the mature islets of the adult organism first become apparent at embryonic day (e) 13.5 and continue to form into early postnatal stages [2]. Committed endocrine cells subsequently differentiate to become five different hormone-producing cell types (β, α, δ, ε, and PP cells). In the early stages of islet morphogenesis (around e17.5), these hormone-expressing endocrine cells can be found in clusters closely associated with the pancreatic ductal epithelium from which they originate [7], [8], [9], [10]. These endocrine clusters must lose their tight association with the ductal epithelium and organize in the acinar parenchyma to form typical islets [7]. The expression of matrix metalloproteinases in endocrine cells (MMPs; known to be involved in extracellular matrix degradation) is thought to aid in the migration of these cells through basement membrane and extracellular matrix [11]. There is also evidence that integrins may be involved in guiding the migration and organization of the endocrine cells, leading to the characteristic islet morphology of an insulin-producing β cell core surrounded by α (glucagon), δ (somatostatin), ε (ghrelin), and PP (pancreatic polypeptide) cells at the periphery. In 2000, Cirulli et al. demonstrated that α_v_β_3_ and α_v_β_5_ integrins could mediate the adhesion and migration of putative endocrine precursors [12]. Despite these and other studies, many of the processes involved in endocrine cell migration/delamination and islet morphogenesis are not yet well understood.

While many of the factors critical for islet morphogenesis remain elusive, a role for cell adhesion molecules has been well described in the formation of morphologically normal pancreatic islets. For example, in transgenic mice, a dominant negative E-cadherin driven specifically to β cells by the rat insulin promoter (RIP) leads to defects in β cell aggregation and islet formation [13]. In contrast, α cells aggregated into clusters lacking β cells. Additionally, in animals with a null mutation in neural cell adhesion molecule (NCAM), islets form with α cells randomly distributed throughout the islet rather than being restricted to the islet periphery [14]. In NCAM mutant islets, the subcellular distribution of epithelial polarity markers was also disrupted, which suggests that NCAM may also be involved in cell polarity and/or adhesion of islet cell types.

In addition to the mouse models mentioned above, transgenic mice over-expressing the cut-homeodomain transcription factor Hepatic nuclear factor 6 (Hnf6) specifically in the pancreatic endocrine cell lineage showed disrupted islet morphogenesis and a form of insulin-dependent diabetes [15]. Defects included aberrant endocrine cell sorting, increased islet size, and failure of islets to migrate away from the ductal epithelium. Due to the fact that these Hnf6 transgenic animals exhibited common features of islet dysmorphogenesis (scattering of islet cell types and abnormal islet size) with the addition of the close apposition of islets to the ductal epithelium, we hypothesized that these animals would serve as a valuable tool in which to identify factors that are critical for normal islet morphogenesis.

In the current study, whole genome microarray analysis was performed on total pancreatic RNA to identify differences in the gene expression profiles of wild type (WT) and Hnf6 transgenic animals at late gestation (e18.5) and postnatal day one (P1), developmental time points when the events of normal islet morphogenesis are occurring [1]. Hnf6 transcription is dramatically down-regulated in endocrine cells of WT animals at this time and the protein is no longer detectable (Tweedie Ables et.al., submitted), thus magnifying the difference in Hnf6 activity for the study. Additionally, these time points are well before the onset of diabetes in the Hnf6 Tg mice ([15] and our unpublished observations), and as such are not susceptible to alterations in gene expression that are common to hyperglycemic animals (reviewed in [16]). We provide the list of genes with altered pancreatic expression in response to islet-specific HNF6 over-expression. We discuss several genes that may be important for normal islet morphogenesis and proper islet function.

## Results and Discussion

### Pdx1^PB^Hnf6 transgenic mice as a model of islet dysmorphogenesis

Islet morphogenesis in the mouse begins around e17.5 and continues postnatally. The formation of islets with the stereotypical architecture of a β cell core surrounded by α, δ, ε and PP cells is thought to be critical for the proper function of pancreatic islets [17], [18], [19], [20], [21]. Despite the fact that many transcription factors important for pancreas/islet development have been identified, most of the factors critical for proper islet morphogenesis and function remain uncharacterized.

**Figure 1:**
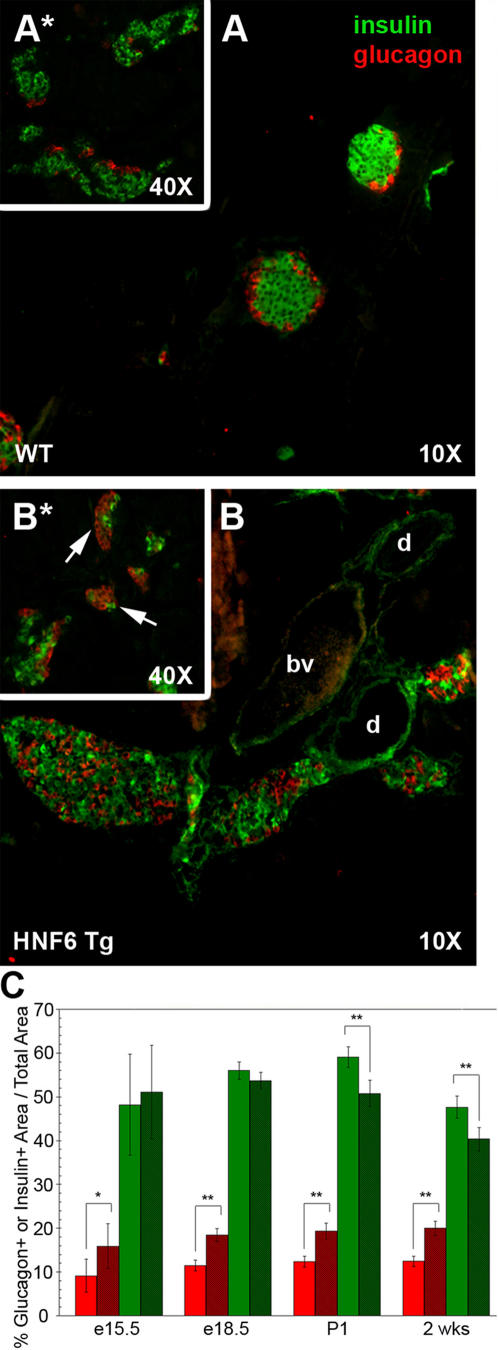
Hnf6 Tg animals exhibit abnormal islet morphogenesis. (A) Islet from a four week old WT animal showing insulin-producing β cells at the islet core (green) and glucagon-producing α cells at the periphery (red). (B) At the same age, individual Hnf6 Tg islets are larger, have a mixed islet phenotype, and are closely apposed to the ductal epithelium. (A*, B*) At e18.5, WT endocrine cells have begun to adopt the stereotypic architecture; however, Hnf6 Tg α cells are often clustered together in islet-like structures that have few, if any, β cells (arrows in B*). (C) Morphometric analysis demonstrates increased glucagon^+^ cell area (red) as early as e15.5 in Hnf6 Tg islets (dark red). This relative increase over WT islets (solid bars) persists into postnatal stages. Total insulin^+^ area decreases in transgenic animals (dark green) at postnatal stages, but does not appear to be altered during embryogenesis. e15.5, n = 4; e18.5, n = 4; P1, n = 2; 2 weeks, n = 3. Error bars were determined by 95% confidence interval. *p<0.05, **p<0.005, as determined by Student's t-test. *d*, duct; *bv*, blood vessel.

We previously described a transgenic mouse model which displayed alterations in islet morphogenesis [15], [22]. In this model, a 1.0 kb endocrine-specific enhancer (PB; [23]) from the 5′ regulatory region of the *pancreas and duodenal homeobox 1* (*Pdx1*) gene was used to drive the expression of Hnf6 specifically to the pancreatic endocrine lineage. *Pdx1* is normally expressed in the antral stomach, pancreas, and proximal duodenum and is essential for pancreas development and mature β cell function [24], [25], [26], [27]. As Hnf6 is normally down-regulated in the endocrine lineage prior to birth, the *Pdx1* ^PB^ *Hnf6* transgene (Hnf6 Tg) allowed for the maintenance of Hnf6 expression in endocrine tissue during late embryogenesis and continuing into adulthood [15]. We found that maintaining Hnf6 expression in islets resulted in a host of morphological and functional deficiencies, including a scattering of cell types within the islets, abnormal islet size, decreased insulin secretion, and a block to β cell maturation (Figure 1; [15], [22]). Additional morphometric analysis during embryonic and perinatal development showed that islet composition is also altered in Hnf6 Tg mice. As seen in Figure 1C, glucagon^+^ cell area was increased in Hnf6 Tg embryos as early as e15.5, and this increase was maintained postnatally (Figure 1C and data not shown). The increase in glucagon^+^ cell area was manifested by larger glucagon^+^ cell clusters that appeared to be segregated from insulin^+^ cells (Figure 1B*). Insulin^+^ area was not affected by the maintenance of Hnf6 until postnatal stages, at which point a significant decrease in insulin^+^ area as compared to WT controls is observed (Figure 1C). As we have previously shown, however, this mild decrease in the number of β cells does not lead to the diabetes observed in this transgenic line [22]. Rather, defects in insulin granule biosynthesis and vesicle trafficking cause the dramatic decrease in glucose stimulated insulin secretion and ultimately, diabetes [22]. The time period of embryonic glucagon^+^ cell population expansion coincides with previous reports of a dramatic increase in hormone-expressing cells in normal pancreas tissue known as the secondary transition, in which pro-endocrine cells that will mature to form the functional units of the adult pancreas are thought to undergo substantial differentiation and proliferation (for review, see [2]).

Since Hnf6 Tg animals exhibited islet dysmorphogenesis, we reasoned that they would serve as an ideal model for the identification of factors important for normal islet morphogenesis. To gain a better understanding of the genes and genetic networks that may underlie the processes of islet morphogenesis and function, we performed a genome-wide microarray analysis, profiling WT whole pancreas tissue versus whole pancreas collected from Hnf6 Tg animals at late embryogenesis (e18.5) and early postnatal (P1) stages. Whole pancreatic RNA has been used by other investigators to examine changes in islet gene expression at these same developmental stages [28]. These are critical times in mouse islet development in which clusters of hormone^+^ cells can be observed to undergo a switch from being randomly assorted to assuming the characteristic islet architecture.

### Characterization of Gene Alterations in Hnf6 Tg animals

Gene expression profiles of WT and Hnf6 Tg pancreata at e18.5 and P1 were generated using the Affymetrix GeneChip Mouse Genome 430 2.0 array (a list of all raw data and supplemental figures can be found at www.vmsr.net/supl/mouse_islet). In addition, the data discussed in this publication have been deposited in NCBIs Gene Expression Omnibus (GEO, http://www.ncbi.nlm.nih.gov/geo/) and are accessible through GEO Series GSE4926. All statistical analysis was performed using GeneSpring 7.0 (Agilent Technologies). Since we were interested in identifying transcripts differentially expressed due to their direct or indirect involvement in islet morphogenesis, RNA isolated from whole pancreas tissue was pooled (3–5 animals per pool) to minimize differences associated with biological variability among samples. To provide a robust technical assessment, this pool was assayed in triplicate (see Methods). Technical replication allows one to evaluate commonalities in gene expression changes and does not reveal differences in gene expression that may be due to inherent biological variability between individual animals, having nothing to do with the genetic manipulation. Our initial observations were subsequently validated independently on individual samples for several of the gene products showing altered expression in the pooled population (see below).

**Figure 2:**
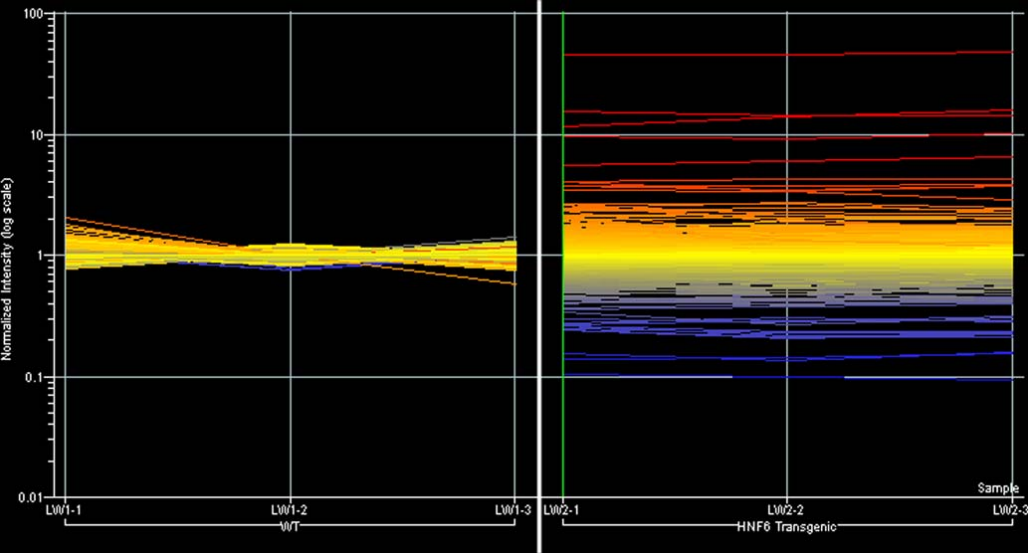
Assessment of replicate consistency and gene expression changes. Each line on the graph represents an individual spot on the array. Normalized ratio values of the average for the three WT samples (WT1-WT3) were plotted against each individual WT sample (left) and each individual Hnf6 Tg sample (right; Tg1-Tg3). Ratio values indicating no change in gene expression are seen in yellow at a value of 1.0. Transcripts that have an altered expression in Hnf6 Tg animals compared to WT animals are indicated in shades of red (greater than 1.0) or blue (less than 1.0).

Gene expression ratio values were calculated by comparing signal intensity values for each individual sample to the average signal intensity value of the control or WT triplicates. This allows an assessment of the consistency of the replicates and to compare gene expression levels in WT and Hnf6 Tg pancreas at e18.5 (data not shown) and P1 (Figure 2). Ratio values for all WT samples were highly consistent with little sample-to-sample variability (Figure 2, left). In the Hnf6 Tg samples plot (Figure 2, right), the majority of the genes remained centered around a ratio value of 1.0 (indicating no change in expression); however, there were also subsets of genes with ratio values greater than and less than 1.0, shown in red and blue, respectively. These represent genes that have an altered expression in Hnf6 Tg animals compared to WT animals.

As proof of principle, we were able to detect *Hnf6* (*Onecut1*) itself as up-regulated two-fold in the Hnf6 Tg mice as compared to WT mice (Supplementary Material Tables S1 and S2). Since the model used in these studies over-expresses Hnf6 specifically in endocrine tissue and not in other pancreatic cell types, detection of altered *Hnf6* levels in whole pancreas from transgenic animals was crucial. These data suggested to us that we were able to detect changes in endocrine-specific gene expression using our experimental design. Since *Hnf6* levels were elevated only twice that of WT levels, it appears that *Hnf6* was not over-expressed at a super-physiological level. However, since the islet cell population represents only about 20–30% of the total pancreatic mass at the stages examined, it could be that the increase in *Hnf6* expression within the islets is actually higher than two-fold. The identification of changes in expression of endocrine genes including *Hnf6* was critical to our analysis because we were interested in understanding changes occurring within the islet cell population. Thus, we are confident that we are able to detect changes occurring within a relatively underrepresented cell type in the pancreas. We note that not all of the observed changes in gene expression are likely to have occurred solely within the endocrine tissue and acknowledge that there could be secondary changes in acinar and ductal gene expression due to over-expression of *Hnf6* in islets.

To detect statistically significant differences in expression levels between the WT and Hnf6 Tg pancreata, we performed t-tests, using the Benjamini and Hochberg multiple testing correction to improve the false discovery rate (the expected proportion of false positives among the results. Lists of genes with a *p*-value of less than 0.05 after this analysis were constructed (Supplementary Material Table S1). A total of 1176 probesets were deemed statistically significant by these criteria in the e18.5 data set along with 484 probesets in the P1 data set. Importantly, genes of particular interest to our study passed the test for statistically significant differential expression. *Onecut1* and its direct transcriptional target *Ngn3* [29] are both included in the t-test results and thus showed a statistically significant difference in expression in the Hnf6 Tg animals compared to WT, as did all of the transcripts discussed in the following sections.

### Microarray Validation

**Figure 3:**
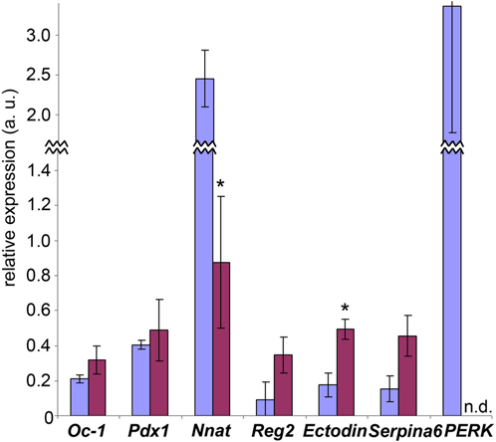
RT-PCR validation of microarray results. P1 pancreatic WT (blue) and Hnf6 Tg (purple) RNA was analyzed for the expression of the transcripts indicated. Values are expressed in arbitrary units as a ratio (transcript:tubulin) of the average of individual samples for each genotype. Error bars represent SEM; *p<0.05. *n.d.*, not detectable. For comparison, microarray analysis of these same transcripts revealed the following: OC-1 increased two-fold; Pdx1 no change; Nnat decreased two-fold; Reg2 increased 1.7-fold; Ectodin increased 1.6- to 1.9-fold; Serpina6 increased 4.6-fold; PERK decreased two-fold.

In order to validate results obtained using the microarray, we chose several candidate genes for RT-PCR analysis. In these studies, we used individual biological samples to confirm the changes observed with technical replicates used for our microarray studies. For validation purposes, we chose to examine several transcripts that we considered potential factors involved in pancreas development, but that have not been fully characterized (*Neuronatin*, *Reg2*, *Ectodin*, and *Serpina6*), as well as transcripts that were important for validation of our model (*Hnf6*, *Pdx1*). As described, the model of islet dysmorphogenesis used for these studies was islet-specific Hnf6 transgenic over-expression. Levels of *Hnf6* (*Onecut1*) were up-regulated at e18.5 and P1 by two-fold in Hnf6 Tg pancreata on the microarray (Supplementary Material Table S2B, D). Using RT-PCR on individual WT and Hnf6 Tg pancreatic RNA extracts, we were also able to detect an increase in *Hnf6* transcript, albeit at slightly lower levels (Figure 3). As Hnf6 has been reported to directly activate *Pdx1* early in pancreas development, we predicted we might detect increased levels of *Pdx1* in Hnf6 Tg animals on the microarray. No change in *Pdx1* expression was detected on the microarray and these results were confirmed with RT-PCR.

*Neuronatin* (*Nnat*), a downstream target of the endocrine cell-enriched transcription factor NeuroD/Beta2 was down-regulated two-fold in Hnf6 Tg samples on the microarray**.** RT-PCR validation on individual pancreatic RNA samples confirmed the down-regulation of *Nnat* observed in Hnf6 Tg animals on the microarray (Figure 3). Nnat is reported to be enriched in the β cell line βTC1, and knock-down of Nnat in cell culture leads to decreased insulin secretion in response to glucose [30], [31]. However, the *in vivo* role for Nnat in pancreas development and endocrine function remains unknown. Our results suggest a role for Nnat in normal endocrine cell development.

*Reg2* (*Regenerating gene 2*) belongs to the mitogenic Reg gene family. In the pancreas, *Reg2* has been identified in acinar tissue and in hyperplastic islets [32]. Up-regulation of *Reg2* has been associated with diet-induced obesity, diabetes and regeneration after partial pancreatectomy, and has also been suggested as a compensatory mechanism for stimulating the proliferation of pancreatic β cells and enhancing insulin secretion [33], [34]. The role of *Reg2* in normal pancreas development has not been investigated. Since Hnf6 animals have larger islets and overt diabetes, we were interested in the up-regulation of *Reg2* in Hnf6 Tg animals observed on the microarray (1.7-fold at P1). RT-PCR analysis confirmed the up-regulation of *Reg2* which was observed in Hnf6 Tg animals via microarray analysis (Figure 3).

Recent studies have shown that BMP signaling augments insulin secretion from pancreatic β cells [35]. As Hnf6 Tg animals have severe defects in insulin secretion, the observed up-regulation of the secreted BMP inhibitor, *Ectodin*, on the microarray (1.6-fold at e18.5; 1.9-fold at P1) was of interest. Since the role of BMP signaling in islet function has only recently been appreciated, *Ectodin* has not yet been studied within the context of islet function. RT-PCR was used to confirm the up-regulation of *Ectodin* in Hnf6 Tg pancreata that was analyzed on the microarray (Figure 3). Given its role as a BMP antagonist in other systems [36], [37], Ectodin should be investigated regarding its role in pancreatic function.

Based on our interest in genes that may be involved in normal islet morphogenesis, we chose to validate the alteration of *Serpina6*, a member of the serine protease inhibitor family. Serpins are involved in maintaining the integrity of the extracellular matrix (ECM). ECM degradation and repair influence cell adhesion and migration, all thought to be critical in islet morphogenesis. Its role in the pancreas has not yet been described. Microarray analysis, confirmed by RT-PCR (Figure 3), revealed that Serpina6 was up-regulated 4.6-fold at P1 in Hnf6 Tg animals compared to WT.

EIF2A3 kinase (*Perk*) is involved in inhibition of protein synthesis, and is involved in the ER stress response in pancreatic β cells [38], [39]. Studies have shown that *Perk* is expressed in β cells and is specifically required in β cells during the fetal and early neonatal period as a prerequisite for normal postnatal glucose homeostasis [40]. Thus, as Hnf6 Tg animals have severe defects in glucose homeostasis [22], it was of interest that microarray analysis showed a two-fold down-regulation of PERK in these animals. Validation of *Perk* down-regulation using RT-PCR showed non-detectable levels of *Perk* in Hnf6 Tg animals, with much higher levels in WT pancreata (Figure 3).

### Gene Ontology Analysis

Gene ontology analysis was performed to classify genes differentially expressed between the WT and Hnf6 Tg pancreata (see Methods). Given the moderate sample size of the test groups (n = 3 per genotype) and the fact that low-abundance, differentially expressed transcripts may be unable to reach significance without additional replicates, the analyzed gene list was broadened to encompass all genes showing at least a 1.5 fold-change in 2 of the 3 Hnf6 Tg replicate samples (Supplementary Material Table S2). This allowed inclusion of candidate genes that did not reach statistical significance but still showed a trend toward differential expression between the WT and Hnf6 Tg samples. By this criterion, a total of 1255 up-regulated probesets and 707 down-regulated probesets were identified when both age groups were considered. Of this gene set, 35 of the down-regulated genes were common to both the e18.5 and P1 analysis, and 41 of the up-regulated genes were common to both ages. A list of the genes common to both ages can be found in Supplemental Material (Table S3). The biological relevance of these trends in gene expression changes will obviously need to be tested on an individual basis. The only transcript of obvious significance to pancreas development in this overlapping dataset was GATA4, a known endodermal transcription factor, which was up-regulated in the transgenic pancreata at both time points examined (2-fold at e18.5; 1.5-fold at P1). Overall, the categories represented by altered genes were congruent with the phenotypic alterations seen in Hnf6 Tg animals [15], [22].

**Figure 4:**
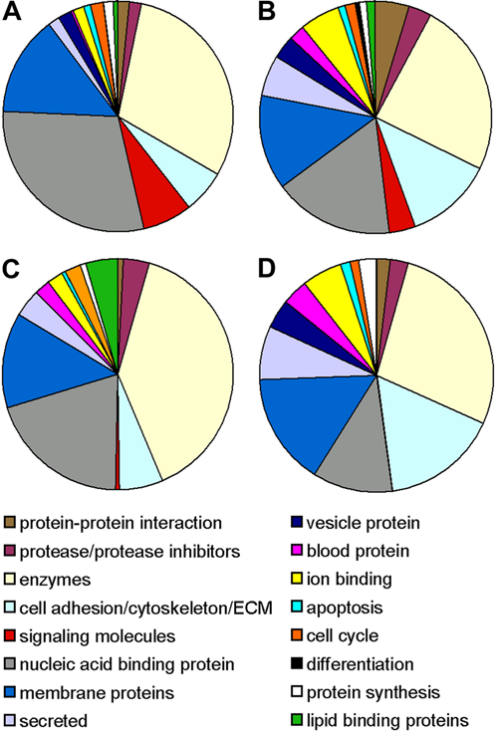
Relative proportion of transcripts altered in Hnf6 Tg animals as determined by gene ontology analysis. Known transcripts altered by 1.5-fold or greater were placed into one of 16 categories, represented by specific colors on each chart. (A) Transcripts up-regulated at e18.5 (total 582). (B) Transcripts down-regulated at e18.5 (total 283). (C) Transcripts up-regulated at P1 (total 135). (D) Transcripts down-regulated at P1 (total 161).

Each gene was placed into one of 17 categories, which include: protease/protease inhibitors, enzymes, cell adhesion, cytoskeletal and ECM, signaling molecules, nucleic acid binding proteins, membrane proteins, secreted proteins, vesicle proteins, blood proteins, and cell cycle (Figure 4). Unknown genes were not included in the analysis but comprised less than 40% of up or down-regulated genes at either time point (unknown genes represent a total of 39.5% of the 45,070 probesets on the microarray). Since the completion of our original analysis, some of these previously “unknown” gene products may subsequently have been functionally characterized and ascribed to a particular cellular process. These transcripts are also included in the entire data set and may be obtained at www.vmsr.net/supl/mouse_islet.

At both time-points (e18.5 and P1), enzymes represented the largest percentage of each group of altered genes (both up- and down-regulated). This is representative of the complete probeset present on the array, as enzymes constitute the largest group of annotated genes (21.2% of 27,260). Based on the dysmorphic phenotype of the Hnf6 Tg islets, we expected to see alterations in factors involved in cell adhesion, cytoskeletal/ECM molecules, and protease/protease inhibitors. These factors are known to be involved in various aspects of islet morphogenesis, including endocrine cell organization, degradation of basement membrane, and delamination of endocrine cells away from ductal tissue [7], [9], [10], [11], [12], [41]. Indeed, we found that 18% and 22% of genes altered at e18.5 and P1, respectively, can be categorized as cell adhesion/cytoskeleton/ECM. In contrast, cell adhesion/cytoskeleton/ECM genes represent only 8.8% of annotated genes on the array, suggesting that these genes in particular are up-regulated in HNF6 transgenic pancreata.

#### Transcription factors

**Figure 5:**
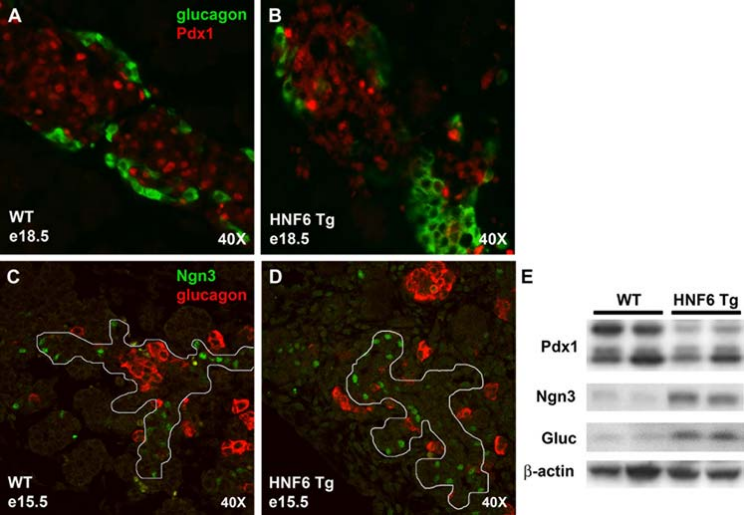
Immunofluorescent and western blot validation of microarray results for Hnf6 transcriptional targets. No changes in Pdx1 expression (red) were observed by co-immunohistochemistry in e18.5 WT (A) and Hnf6 Tg (B) embryonic pancreata (glucagon shown in green). In contrast, Increased numbers of Ngn3^+^ cells (green) were observed within the pancreatic epithelium (outlined) at e15.5 (glucagon^+^ cells shown in red) in HNF6 Tg pancreata (C) as compared to WT pancreata (D). P1 pancreatic extracts (E) were probed for the expression of the proteins indicated. Values are expressed as a ratio (WT:Hnf6 Tg). Two representative samples for each genotype are shown.

Approximately 20% of all annotated transcripts that showed altered expression on the microarray were categorized as nucleic acid binding proteins (Figure 4). This is representative of the complete annotated probeset, in which 19.6% of all transcripts encode nucleic acid binding proteins. One transcript of interest, the pro-endocrine transcription factor *Ngn3 (Neurog3)*, was up-regulated two-fold at e18.5 by microarray analysis (Supplementary Material Tables S1B, S2B). Lineage tracing [42] and global deletion analyses [43] have shown that *Ngn3* is required for the differentiation of all endocrine cells in the pancreas, and as such is one of the earliest transcription factors that specifically marks the endocrine population prior to cell type-specific hormone expression [44]. Previous research has shown that *Ngn3* is a direct target of Hnf6 transcriptional activity; global *Hnf6* ^−/−^ mice have a dramatic down-regulation of *Ngn3* ^+^ cells [29]. Furthermore, over-expression of Ngn3 within the Pdx1^+^ domain results in an expansion of the endocrine population, specifically in glucagon-producing cells [44]. Thus, the increased numbers of glucagon^+^ cells we observed in Hnf6 Tg pancreata at e15.5, e18.5, and P1 (Figures 1C and 5B, E) may be due, in part, to increased expression of *Ngn3*. We have used both western blot and immunohistochemistry analyses to validate the up-regulation of *Ngn3* transcripts in Hnf6 Tg pancreata (Figure 5). These experiments show that the number of Ngn3^+^ cells at e15.5 and the amount of Ngn3 protein at P1 (1.2 fold increase in Tg) is increased in Hnf6 Tg pancreata (Figures 5C–E). This data correlates well with the up-regulation of *Ngn3* in the e18.5 microarray data set (Supplementary Material Table S2B). Thus, over-expression of Hnf6 specifically in the endocrine lineage results either in an increase in the absolute number of *Ngn3* ^+^ endocrine progenitor cells, or increases the duration of NGN3 expression in these cells. Regardless of the mechanism, the effects of HNF6 over-expression on *Ngn3* expression are transient, as microarray analysis at P1 failed to show an increase in *Ngn3* expression (Supplementary Material Table S2D).

Microarray analysis of Hnf6 Tg animals also revealed a 1.7-fold increase in the expression of the paired-homeodomain transcription factor *Pax6* (Supplementary Material Table S2B). Pax6 is known to be important for normal α cell development/number in the pancreas [45]. Therefore, its up-regulation in the Hnf6 Tg animals is not entirely surprising due to the fact that both α cell number and glucagon protein levels are increased in these animals at the time when the microarray analysis was performed (Figures 1C and 5B). *Pax6* has also been shown to be altered in another study looking at Hnf6 transcriptional regulation using rat insulinoma cells [46]; see *Conclusions*). This may suggest that *Pax6* is in some capacity regulated (directly or indirectly) by Hnf6 in the rodent pancreas.

As a transcriptional activator, Hnf6 has been proposed to directly transactivate *Pdx1* [47]; however, microarray analysis of Hnf6 Tg animals did not show a change in the expression of *Pdx1*. Consistent with these results, immunohistochemistry for Pdx1 at e18.5 (Figure 5A–B), failed to detect significant changes in the number of Pdx1^+^ cells in Hnf6 Tg pancreata. Likewise, no significant alterations in the level of Pdx1 protein at P1 were found by western blot analysis (Figure 5E). While we hypothesized that as a direct activator of *Pdx1*, over-expression of Hnf6 could lead to an increase in the number of Pdx1^+^ cells similar to that seen for Ngn3, our results are indeed consistent with mouse models of Hnf6 loss-of-function. A previous report in the literature and additional evidence from our lab has shown that early expression of Pdx1 is delayed in models where *Hnf6* expression is decreased or absent, but Pdx1 expression levels normalize later in development [47]; Zhang et. al., submitted). These results suggest that Hnf6 may positively regulate *Pdx1* gene expression at early developmental stages, but not at the stages used in our analyses. Alternatively, the islet-specific *pdx1* promoter fragment used to drive the transgene may compete with endogenous regulators of *pdx1* gene expression, thus dampening the effect of HNF6 over-expression on the endogenous *pdx1* locus.

#### Cell adhesion and migration

Based on the fact that islets over-expressing Hnf6 do not properly separate from ducts or incorporate into surrounding acinar tissue, we were interested in factors involved in cellular adhesion and migration and the coordinated remodeling of extracellular matrix (ECM). Of particular interest for this study were cell adhesion/migration factors and/or proteases/protease inhibitors that were down-regulated in the Hnf6 Tg animals. This may suggest that one or more of these molecules are critical for the “movement” of islet structures away from the ducts during islet morphogenesis, and its down-regulation caused a disruption in the process. At P1 six cell adhesion molecules were down-regulated, and one cell adhesion molecule was up-regulated. Down-regulated cytoskeletal genes include myosin, and kinesin family members.

Interestingly, several factors with altered expression in Hnf6 Tg animals had previously been reported to be involved in modulating cell adhesion and migration or remodeling of the ECM. Such molecules include: *Matrix metalloproteinases* (*MMP*) *8* (1.7-fold down-regulated at e18.5) and *14* (1.6-fold up-regulated at e18.5), and the protease inhibitors *Timp3* (1.9-fold up-regulated at e18.5) and *Serpina1* (1.5-fold down-regulated at e18.5), all known to be involved in the remodeling of extracellular matrix; and *A disintegrin and metalloprotease* (*ADAM*) family members *10* (1.6-fold up-regulated at e18.5) and *33* (1.7-fold down-regulated at e18.5). Proteases and protease inhibitors comprise only 0.9% of all annotated sequences of the array, while they represent: 3.5% of genes down-regulated at e18.5, 1.7% of genes up-regulated at e18.5, 2.5% of genes down-regulated at P1, and 3.7% of genes up-regulated at P1. Thus, as with cell adhesion and ECM molecules, these types of genes are specifically increased by HNF6 over-expression.

*Connective tissue growth factor* (*CTGF*), a secreted factor which binds to integrins and elicits a variety of biological responses in various tissues, including cell migration [48] was down-regulated 2.5-fold at P1. Preliminary data from our laboratory indicates that loss of CTGF results in defects in islet morphogenesis similar to those seen in the HNF6 Tg animals, and decreased embryonic β cell proliferation (Crawford et. al. submitted).

We also identified many genes that have been characterized to be involved in morphogenetic processes in neurons. Pancreatic endocrine cells have many molecular similarities with neuronal cell types, such as: 1) developmental requirement for the same transcription factors or closely related family members [43], [49], [50], [51], [52], [53], [54]; 2) involvement of the Notch signaling pathway in progenitor allocation [3]; and 3) the deposition of amyloid in disease states such as Alzheimer's and Type 2 diabetes [55]. Examples of genes altered in the Hnf6 Tg animals that have been implicated in cell migration in the brain include: *Abi2* (1.7-fold down-regulated at e18.5), an adaptor protein which, when inactivated, results in abnormal lens fiber migration in the eye (Supplementary Material Table S2A; [56]); *Stathmin* (*Stmn*) (∼1.7-fold down-regulated at e18.5 and P1), a microtubule-associated protein whose over-expression leads to increased motility of GnRH neurons (Supplementary Material Table S2A; [57]); and *Neogenin* (*Neo1*) (1.9-fold down-regulated at e18.5), a protein expressed in the extending fiber cells of the developing lens, implicating it in migration events of these fibers (Supplementary Material Table S2A; [58]). *Neogenin* has also recently been reported to be localized to the developing pancreatic epithelium, strengthening the possibility of its involvement in pancreatic islet morphogenesis [59].

#### Cell proliferation

Prompted by reports regarding the role of Hnf6 in the proliferation of hepatocytes [60] and the binding of Hnf6 to the proximal promoter regions of several known cell cycle regulators in both liver and pancreas [61], we examined our microarray data for changes in the expression of cell cycle genes in Hnf6 Tg animals. Interestingly, we found that cell cycle genes were more likely to be up-regulated by 1.5-fold or higher at e18.5 and P1 than down-regulated (2.2% up-regulated at both e18.5 and P1; 1.4% and 1.2% down-regulated at e18.5 and P1, respectively). We detected alterations in several known cell cycle regulators, many of which have been implicated in postnatal β cell replication: *Cyclin D1* (1.8-fold up-regulated at e18.5), *Cyclin D2* (two-fold up-regulated at e18.5), *Cyclin G1* (1.5-fold up-regulated at e18.5), and *Cyclin B2* (1.6-fold up-regulated at P1); *Menin* (1.7-fold up-regulated at e18.5); *p21* (1.9-fold up-regulated at e18.5); and *p57* (1.5-fold down-regulated at P1) (Supplementary Material Table S2; [62], [63], [64], [65]). Thus, we have provided evidence that several cell cycle regulators are impacted by the maintained expression of Hnf6. This correlates well with previous reports of Hnf6 interaction with cell cycle genes, such as the key cell cycle regulator FoxM1 [60].

**Figure 6:**
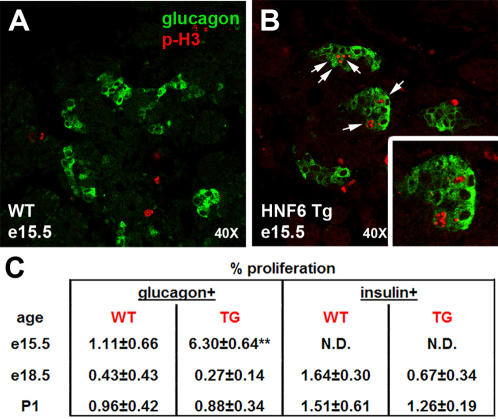
Increased proliferation of glucagon^+^ cells in Hnf6 Tg pancreata. Proliferation rates in WT (A) versus Hnf6 Tg (B) pancreata were determined by co-immunohistochemistry for phosphorylated histone H3 (p-H3, red) and either glucagon (green) or insulin (data not shown). (C) Number of cells that co-expressed p-H3 and either glucagon or insulin are expressed as a percentage of the total hormone^+^ cells counted at e15.5, e18.5, and P1; **p = 0.004.

We hypothesized that increased proliferation of glucagon^+^ cells could contribute in part to the increase in this cell type. As significant increases in the number of hormone^+^ cells can normally be detected between e14.5 and e16.5 [2], we analyzed pancreata from e15.5 embryos for co-localization of either glucagon or insulin with phosphorylated histone H3 (p-H3), a marker of mitosis, by immunofluorescence (Figure 6). Indeed, there was a significant increase in glucagon^+^/p-H3^+^ cells in Hnf6 Tg embryos as compared to WT littermates, while no difference was detected in proliferation of insulin^+^ cells at this time. No significant changes in proliferation could be detected in glucagon^+^ or insulin^+^ cells at e18.5 or P1 (Figure 6C).

#### Insulin granule biosynthesis and secretion

In order to secrete insulin in response to changes in blood glucose concentration, β cells are highly specialized to coordinate protein synthesis and modification with Ca^2+^-sensitive regulated exocytosis (for review, see [66], [67]. To this end, a readily-releasable pool of insulin protein is stored in the cytoplasm within dense-core secretory vesicles (“granules”). Upon glucose stimulation, membrane depolarization by K^+^-ATP channels triggers an influx in Ca^2+^ via L-type Ca^2+^ channels. Proteins such as synaptotagmins are then thought to act as Ca^2+^ sensors that initiate the assembly of the basic fusion machinery as well as the mobilization of insulin granules (“priming”) for exocytosis.

We previously showed that Hnf6 Tg animals have impaired glucose-stimulated insulin secretion (GSIS) and post-weaning hyperglycemia, as well as significant decreases in levels of the glucose transporter GLUT2 [15]. Subsequent analyses revealed that defects in insulin secretion may be attributable to altered insulin granule maturation, trafficking, and release [22]. Most strikingly, β cells from Hnf6 Tg animals at six weeks of age have a dramatic reduction in all insulin granules, correlating with a loss of secretagogue-stimulated insulin secretion. These cellular alterations are consistent with late postnatal alterations in protein trafficking, either within the trans-golgi network (TGN) or in the maturation, shuttling, and ultimate exocytosis of insulin granules from the β cell.

Microarray analysis at both e18.5 and P1 showed alterations in gene expression at all levels of insulin exocytosis, including: protein synthesis, protein modification and packaging, vesicle maturation, granule exocytosis, and post-exocytosis vesicle recycling. One example, the *Vacuolar-type H^+^-ATPase* (*V-ATPase* or *ATP6V0A1*; 1.8-fold up-regulated at e18.5), localized to both the TGN and granules in secretory cells, is thought to provide the proper pH for processing and acidification/maturation of insulin granules (Supplementary Material Table S2B; [68]). Several transcripts altered in our analysis, including the granule-localized *Granuphilin* (1.5-fold down-regulated at P1), *Cellubrevin* (*VAMP3*) (1.6-fold up-regulated at e18.5), *Rab3d* (1.6-fold up-regulated at e18.5), and *Rab27a/b* (1.7-fold up-regulated at e18.5), have each been implicated in granule fusion to the plasma membrane in preparation for exocytosis [69], [70], [71], [72], [73]. Similarly, the plasma membrane-localized *Synaptotagmin 7*, down-regulated 1.5-fold at P1, is thought to be important for targeting exocytosis machinery to specific localization points on the plasma membrane (reviewed in [67]). Furthermore, both *Rab3a* and *Rab27a/b* appear to play important roles specifically in β cell regulated exocytosis [73], [74]. The Rab family members are GTP-binding proteins that appear to function as regulators of the secretory and endocytic pathways. *Rab3* and *27* are predominately localized to granules of neuroendocrine secretory cells, and use effector proteins such as Granuphilin to move granules via the unconventional Myosin Va (down-regulated 1.5-fold at e18.5) along the actin cytoskeleton to the plasma membrane for exocytosis (Supplementary Material Table S2; [69], [75], [76]). Lastly, the CDK5 inhibitor *CDK5RAP1* (C42; up-regulated 2.1-fold at P1) has also been recently implicated in the regulation of this process (Supplementary Material Table S2D; [77], [78], [79]), though a thorough *in vivo* analysis of this inhibitor's role in insulin granule exocytosis has not yet been performed.

Although it is still not known whether any of these genes are direct targets of Hnf6, Hnf6 Tg mice may provide a new model for understanding how changes at the transcriptional level can alter the processing and exocytosis of insulin, and how these defects ultimately lead to changes at the physiological level (i.e., diabetes).

### Conclusions

The prospect of generating optimally functioning islets *in vitro* will likely rely on a complete understanding of how transcription factor networks and cell interactions regulate proliferation, differentiation, and morphogenesis of normal pancreatic endocrine cells. Although many transcription factors critical for these processes have been identified and characterized, information about genes involved in islet morphogenesis has remained rather elusive. In order to take an unbiased approach to the identification of previously unappreciated islet morphogenetic factors, we genetically profiled a model of islet morphogenesis from our laboratory. The Hnf6 Tg animals were previously reported to have diabetes and islet dysmorphogenesis, characterized by a mixed islet phenotype and islets with close apposition to ducts [15]. Since microarray analysis generates vast amounts of data, the resulting data were categorized based on the functional role of each altered transcript. Of primary interest to us were genes involved in the processes predicted to be critical for islet morphogenesis (cell adhesion, cell communication, cell migration, ECM degradation, and cell sorting). In fact, we found that these classes of genes were disproportionately represented in our list of altered genes when compared with their representation in the total probeset present on the microarray.

There have been few studies addressing potential transcriptional targets of Hnf6 in the pancreas. In one study, Odom and colleagues constructed custom DNA microarrays targeting genomic regions located 700 base pairs upstream and 200 base pairs downstream of transcription start sites [61]. They subsequently performed chromatin immunoprecipitation combined with promoter microarrays in order to identify genes occupied by Hnf6 in isolated adult human pancreatic islets. The primary interest in the present study was not to identify direct binding targets of Hnf6, but rather to use the Hnf6 Tg animals as a tool for the identification of genes important for islet morphogenesis. Although the two studies are quite different in nature, comparison of the data sets reveals 16 genes in common between the two studies as well as similar, but not identical, gene products. For example, *Connexin32*, *Hnf4*, and *Meis1* as well as *Claudin*, *Serpin*, and Sec-like family members were identified in both studies (Supplementary Material Table S2; [61]). Therefore, the information provided in the Odom study suggests that these genes are also direct targets of Hnf6 during islet morphogenesis (e18.5 or P1). The remaining genes identified in the present study, but not found in the Odom analysis, are likely to be a combination of 1. indirect targets, 2. direct targets where Hnf6 binds outside the proximal promoter region, 3. genes that are direct Hnf6 targets during islet morphogenesis but are not expressed in the adult cultured islets, and 4. genes that are secondarily altered due to changes in non-endocrine cells within Hnf6 transgenic pancreata.

In another study, an inducible *Hnf6* transgene was introduced into INS-1 cells [46]. Microarray analysis of INS-1 cells induced with tetracycline to express HNF6 revealed changes in gene expression patterns in some genes in common with our analysis. Genes common to both studies include: *Onecut1* (*Hnf6*), *Met*, *Ddc*, *C1galt1*, *Dig1*, *Iapp*, *Pax6*, *Gnas*, and *Ghr* (Supplementary Material Table S2; [46]. It is not surprising that there are only a small subset of overlapping genes between our analysis and the Thomas analysis, due to some major differences in study design. For example, our study used a mouse Affymetrix chip with RNA from *in vivo* whole mouse pancreas, while the Thomas paper used a rat platform with RNA from a pancreatic β cell line (isolated from rat insulinoma). While these studies were performed to reveal different information, comparing data across the three can help to shed new light on the similarities and differences in the numbers and types of genes Hnf6 regulates (directly or indirectly) in different organisms and cell types.

Microarray analysis provides an opportune method for comparing, on a large scale, changes in gene expression that result from single factor manipulations. We hypothesized that using the Hnf6 Tg mouse as a model for islet dysmorphogenesis would provide an excellent tool for identifying new genes involved in the cellular processes that establish endocrine pancreatic morphology and architecture. We have identified a host of new gene targets, many of which have not been characterized in the pancreas or in the process of islet morphogenesis. Using this data, perhaps in combination with information gained from future microarray analyses of other mouse models, may help to dissect and understand the regulatory networks involved in pancreatogenesis, ultimately leading to a complete picture of how islet architecture contributes to the finely tuned function of endocrine cell types.

## Materials and Methods

### Animals

*Hnf6* expression was driven specifically to pancreatic endocrine cells beginning at e11.5 using a 1.0 kb islet enhancer (−2642 to −1654) from the 5′ *Pdx1* mouse promoter region as previously described [15]. This transgenic line, termed *Pdx1* ^PB^ *Hnf6*, was maintained on an inbred hybrid (B6D2) background. All mice were kept on a 12 hour light/dark cycle, fed Mouse diet 5015 (LabDiet), and given water ad lib. For embryonic analyses, the morning of the vaginal plug was considered e0.5.

All mouse studies were performed in accordance with the Vanderbilt Institutional Animal Care and Use Committee guidelines under the supervision of the Division of Animal Care.

### Tissue acquisition and RNA isolation

Pancreata were dissected at e18.5 and P1 in ice cold PBS and immediately placed into RNAlater (Ambion). RNA from whole pancreas was isolated using the RNAqueous RNA Isolation Kit (Ambion) according to the manufacturer's instructions. Agilent's Bioanalyzer microfluidic assay (Agilent Technologies, Palo Alto CA) was used to assay RNA integrity (Supplemental Figure S1). Spectrophotometric and fluorometric methods were combined to quantitate protein and nucleic acids present in the sample, and to ensure quality control of each sample. RNA samples were bioanalyzed individually to assess RNA quality and integrity. Highly pure samples were pooled according to their genotype (3-5 animals per pool) in order to obtain an adequate quantity of RNA. Supplemental Figure S1 shows the results for individual and pooled samples at e18.5, but similar results were obtained at P1.

### Microarray Analysis

Following quality control, the RNA was prepared for microarray analysis using the standard Affymetrix protocol (Affymetrix Inc, Santa Clara, CA). Briefly, a total of 5 µg of total RNA was reverse transcribed to double-stranded (ds) cDNA using an oligo-dT primer coupled to a T7 promoter. *In vitro* transcription from the ds cDNA was then carried out using T7 polymerase and incorporating biotin-modified CTP and UTP ribonucleotides. The biotinylated cRNA (15 µg) was fragmented and hybridized in triplicate to Affymetrix GeneChip Mouse Genome 430 2.0 arrays containing 45,000 sets of 11 to 25-mer oligomers, representing 39,000 mouse transcripts (34,000 are annotated as well-defined genes). Hybridized cRNA was detected using streptavidin coupled to phycoerythrin and visualized by the Affymetrix 3000 7G laser scanner. The image data was quantitated to generate gene expression values and ratios of gene expression between the hybridized samples. For statistical analysis, CEL files (raw Affymetrix data) were processed using GeneSpring 7.0 (Silicon Genetics) and normalized using Robust Multi-array Analysis (RMA; [80]). T-tests using the Benjamini and Hochberg multiple testing correction were performed on the data to identify genes differentially expressed between the two groups with a significance score of less than 0.05. Statistically significant differences in gene expression between WT pancreas and HNF6 pancreas tissue were confirmed using RT-PCR, western blot and immunohistochemistry. Gene ontogeny analyses were performed using the annotations provided in the GeneSpring 7.0 program. Gene products not annotated in the GeneSpring program were manually investigated individually using Unigene on the NCBI website (http://www.ncbi.nlm.nih.gov/sites/entrez) to determine whether the gene had been ascribed a particular function or found to be part of a known metabolic pathway.

### Tissue preparation and histology

Digestive organs or isolated pancreata from varying embryonic and adult stages were dissected in PBS and fixed immediately in 4% paraformaldehyde (4°C; 45–60 min). Tissues were dehydrated in an increasing ethanol series followed by two xylene washes, infiltrated with xylene:paraffin (1∶1, v/v) and two changes of paraffin at 56°C, and embedded for sectioning.

Serial 5 µm sections were deparaffinized and rehydrated in a decreasing ethanol series to distilled water. Indirect protein localization was obtained by incubation with the following primary antibodies: guinea pig α-insulin (1/1000, Linco), rabbit anti-glucagon (1/1000, Linco), rabbit anti-phosphohistone H3 (1/50, Upstate Biotech), rabbit anti-Pdx1 (1/1000, a gift of C. Wright, Vanderbilt University), and guinea pig anti-Ngn3 (a gift of M. Sander, UC Irvine). All primary antibodies were incubated overnight at 4°C in a humidified chamber. Detection of phosphohistone H3 (pH3), Pdx1, and Ngn3 required antigen retrieval in either citrate buffer (pH3, Pdx1) or TEG buffer (Ngn3) and previously described [22]. Primary antibodies were detected by species-specific donkey secondary antibodies conjugated with Cy2 or Cy3 fluorophores (1/500, Jackson Immunoresearch). Fluorophores were excited using either an Olympus BX41 research microscope or a Zeiss LSM 510 confocal microscope. TIFF images were captured using MagnaFire software (Optronics). Image tonal range and color balance were minimally adjusted via histogram using Adobe Photoshop. Proliferation studies were performed on a minimum of three individual animals at each time point for each genotype.

### Morphometric analysis of endocrine cell and total islet area

Paraffin sections from two to four animals per genotype per age (at least 150 islets total per category) containing endocrine tissue were chosen at random intervals along the length of the pancreas (see Figure 1 legend for actual numbers). Following immunofluorescent detection of insulin and glucagon, Metamorph morphometric software (Universal Imaging) was used to measure the approximate area on each section occupied by either insulin (green channel) or glucagon (red channel) by manual thresholding of pixel intensity. Data were then expressed as a percentage of insulin^+^ or glucagon^+^ area to the total endocrine area measured. Statistical analysis was performed by two-tailed Student's t-test using Excel software.

### Protein extraction and western blotting

Western blot analysis was used to validate specific microarray candidates in individual pancreatic protein extracts. Embryonic pancreata were dissected and immediately placed into extraction buffer containing a protease inhibitor cocktail (0.5 mg/L TPCK, 0.5 mg/L TLCK, 0.6 µM leupeptin, and 2 µM pepstatin), DTT, and PMSF (detailed protocol provided upon request). Samples were homogenized with a small volume motorized pestle, centrifuged to remove cellular debris, and the supernatant frozen at −80°C for long-term storage. Protein was quantitated by the Bio-Rad DC protein assay according to manufacturer's instructions.

Protein was electrophoresed on either 10–20% Tris-glycine gels or 4–12% Bis-Tris gels under denaturing conditions, and blotted to PVDF membrane using the NuPAGE western blotting system (Invitrogen). To ensure equivalent amounts of total protein were loaded per lane, blots were stained briefly with Ponceau S (Sigma). Blots were then blocked in 5% non-fat milk in TBS (pH 7.6) for one hour at room temperature and probed with the following primary antibodies diluted in 3% non-fat milk in TBS and incubated overnight at 4°C: rabbit anti-Pdx1 (a gift of C. Wright, 1/1000), rabbit anti-Ngn3 (Abcam, 1/1000), goat anti-β-actin (Santa Cruz, 1/5000). Rabbit anti-glucagon (Linco, 1/500) was also used to probe blots, but required blocking in 20% non-fat milk in TBS (pH 7.6) overnight at 4°C, and incubation in primary antibody diluted in 5% non-fat milk for one hour at 4°C. Blots were washed in 0.05% Tween-20 in TBS for 30 minutes at room temperature with three changes of buffer. HRP-conjugated species-specific secondary antibodies were diluted to 1/2000 (anti-goat IgG, Santa Cruz) or 1/5000 (anti-rabbit IgG, Amersham) in 1% non-fat milk in TBS and incubated for one hour at room temperature. Following washes as previously described, protein detection was facilitated by the ECL detection system (Amersham) per manufacturer's instructions using Kodak X-Omat Blue film. Protein levels in individual pancreata were then quantitated on a Molecular Imager FX densitometer (Bio-Rad) using Quantity One 4.6 software (Bio-Rad) and normalized to the quantity of β-actin obtained for each sample. Protein levels are thus illustrated as a ratio of WT:Hnf6 Tg. For Pdx1 the density of all three bands (includes both unmodified and post-translationally modified forms of the protein) was included in the quantitation.

### Semi-quantitative reverse transcriptase PCR

RNA was isolated from individual pancreata at P1 as described above. Samples were DNase treated to remove contaminating genomic DNA per the manufacturer's instructions (Ambion). cDNA synthesis was performed on individual wild type (n = 3–4) and HNF6 Tg (n = 3–4) using Applied Biosystems reverse transcription reagents according to the manufacturer's instructions.

Pancreatic mRNA expression was analyzed by PCR (30 cycles of amplification) using intron-exon boundry spanning primers with the following sequences: PERK fwd: gttgctgattggaaggtcat; PERK rev: agtcgtatttactttcagtct; OC1 fwd: cagcgaccttgcaggaat OC1 rev: gctttcagctttgcctcct; Pdx1 fwd: aagctcacgcgtggaaag; Pdx rev: gccgggagatgtatttgttaaa; Serpina6 fwd: ccctcatcctgatcaactacatc; Serpina6 rev: tcattcacatagaagtcctcctctc; *Reg2* fwd: aattgaagaccgtttgacctg; *Reg2* rev: gacctgcattcatgttctgg; Nnat fwd: cacccactttcggaaccat; Nnat rev: tgcagcattccaggaaca; Ectodin fwd: aacagcaccctgaatcaagc; Ectodin rev: cagcccacttgaactcgac

Transcript levels in individual pancreata were quantitated on a Molecular Imager FX densitometer (Bio-Rad) using Quantity One 4.6 software (Bio-Rad) and normalized to the quantity of tubulin for each sample. All values for individual transcripts were normalized to tubulin expression. Data are expressed as a ratio of transcript to tubulin in arbitrary units.

## Supporting Information

Figure S1 Bioanalysis of individual and pooled RNA samples used for microarray analysis at e18.5. (A) The samples were quantified in 10mM Tris on a Nanodrop ND-1000 Spectrophotometer. In addition to concentration, this instrument reports the 260/280 absorption ratio, which can be an important indicator of hidden contaminants. RNA integrity was assessed on total RNA isolated from individual pancreata at e18.5 using microfluidic analysis. The electropherogram it produces makes it easy to detect certain contaminants or degradation, and to calculate the 28s:18s ratio. Intensity and clarity of 28s and 18s ribosomal RNA bands (indicated) was used as a measure of RNA quality in addition to the lack of degradation products below the 18S band. The far left lane is the molecular weight ladder. Lanes 2, 3, 5, 7, and 8 represent total pancreatic RNA isolated from HNF6 transgenic animals. Lanes 1, 4, 6, and 9 represent total pancreatic RNA isolated from wild type littermates. For microarray analysis, RNA from all transgenic samples were pooled (5 total), while samples 1, 4, and 9 were pooled (3 total) to generate wild type RNA. Sample number 6 was discarded due to the increased presence of degradation products in this sample. (B) Bioanalysis results from the pooled wild type samples (lane 1) and pooled transgenic samples (lane 2). These samples were labeled and used for microarray hybridization. Lane 3 is the molecular weight ladder.
(0.46 MB TIF)

Table S1 Statistically significant transcripts altered in Hnf6 Tg pancreata. Complete list of transcripts down- (A, C) or up-regulated (B, D) at e18.5 (A, B) or P1 (C, D) with a p value of <0.05 as determined by Benjamini and Hochberg (variance unequal) statistical analysis.
(0.38 MB XLS)

Table S2 Transcripts altered by 1.5-fold or greater in Hnf6 Tg pancreata. Complete list of transcripts down- (A, C) or up-regulated (B, D) at e18.5 (A, B) or P1 (C, D) with a change in expression of 1.5-fold or greater.
(0.42 MB XLS)

Table S3 Intersection of transcripts altered at both e18.5 and P1 in Hnf6 Tg pancreata. Down- (A) and up-regulated (B) genes common to both e18.5 and P1 data sets as determined by a change in gene expression of 1.5-fold or greater.
(0.03 MB XLS)

## References

[1] Ryan EA, Paty BW, Senior PA, Bigam D, Alfadhli E (2005). Five-year follow-up after clinical islet transplantation.. Diabetes.

[2] Jensen J (2004). Gene regulatory factors in pancreatic development.. Dev Dyn.

[3] Apelqvist A, Li H, Sommer L, Beatus P, Anderson DJ (1999). Notch signalling controls pancreatic cell differentiation.. Nature.

[4] Murtaugh LC, Stanger BZ, Kwan KM, Melton DA (2003). Notch signaling controls multiple steps of pancreatic differentiation.. Proc Natl Acad Sci U S A.

[5] Hart A, Papadopoulou S, Edlund H (2003). Fgf10 maintains notch activation, stimulates proliferation, and blocks differentiation of pancreatic epithelial cells.. Dev Dyn.

[6] Hald J, Hjorth JP, German MS, Madsen OD, Serup P (2003). Activated Notch1 prevents differentiation of pancreatic acinar cells and attenuate endocrine development.. Dev Biol.

[7] Pictet RL, Clark WR, Williams RH, Rutter WJ (1972). An ultrastructural analysis of the developing embryonic pancreas.. Dev Biol.

[8] Teitelman G, Lee JK (1987). Cell lineage analysis of pancreatic islet development: glucagon and insulin cells arise from catecholaminergic precursors present in the pancreatic duct.. Dev Biol.

[9] Alpert S, Hanahan D, Teitelman G (1988). Hybrid insulin genes reveal a developmental lineage for pancreatic endocrine cells and imply a relationship with neurons.. Cell.

[10] Gu D, Sarvetnick N (1993). Epithelial cell proliferation and islet neogenesis in IFN-g transgenic mice.. Development.

[11] Miralles F, Battelino T, Czernichow P, Scharfmann R (1998). TGF-beta plays a key role in morphogenesis of the pancreatic islets of Langerhans by controlling the activity of the matrix metalloproteinase MMP-2.. J Cell Biol.

[12] Cirulli V, Beattie GM, Klier G, Ellisman M, Ricordi C (2000). Expression and function of alpha(v)beta(3) and alpha(v)beta(5) integrins in the developing pancreas: roles in the adhesion and migration of putative endocrine progenitor cells.. J Cell Biol.

[13] Dahl U, Sjodin A, Semb H (1996). Cadherins regulate aggregation of pancreatic beta-cells in vivo.. Development.

[14] Esni F, Taljedal IB, Perl AK, Cremer H, Christofori G (1999). Neural cell adhesion molecule (N-CAM) is required for cell type segregation and normal ultrastructure in pancreatic islets.. J Cell Biol.

[15] Gannon M, Ray MK, Van Zee K, Rausa F, Costa RH (2000). Persistent expression of HNF6 in islet endocrine cells causes disrupted islet architecture and loss of beta cell function.. Development.

[16] Roche E, Maestre I, Martin F, Fuentes E, Casero J (2000). Nutrient toxicity in pancreatic beta-cell dysfunction.. J Physiol Biochem.

[17] Meissner HP (1976). Electrophysiological evidence for coupling between beta cells of pancreatic islets.. Nature.

[18] Bennett MV, Goodenough DA (1978). Gap junctions, electrotonic coupling, and intercellular communication.. Neurosci Res Program Bull.

[19] Halban PA, Wollheim CB, Blondel B, Meda P, Niesor EN (1982). The possible importance of contact between pancreatic islet cells for the control of insulin release.. Endocrinology.

[20] Bosco D, Orci L, Meda P (1989). Homologous but not heterologous contact increases the insulin secretion of individual pancreatic B-cells.. Exp Cell Res.

[21] Samols E, Stagner JI, Ewart RB, Marks V (1988). The order of islet microvascular cellular perfusion is B––A––D in the perfused rat pancreas.. J Clin Invest.

[22] Tweedie E, Artner I, Crawford L, Poffenberger G, Thorens B (2006). Maintenance of hepatic nuclear factor 6 in postnatal islets impairs terminal differentiation and function of beta-cells.. Diabetes.

[23] Gannon M, Gamer LW, Wright CV (2001). Regulatory regions driving developmental and tissue-specific expression of the essential pancreatic gene pdx1.. Dev Biol.

[24] Jonsson J, Carlsson L, Edlund T, Edlund H (1994). Insulin-promoter-factor 1 is required for pancreas development in mice.. Nature.

[25] Offield MF, Jetton TL, Labosky PA, Ray M, Stein RW (1996). PDX-1 is required for pancreatic outgrowth and differentiation of the rostral duodenum.. Development.

[26] Ahlgren U, Jonsson J, Jonsson L, Simu K, Edlund H (1998). beta-cell-specific inactivation of the mouse Ipf1/Pdx1 gene results in loss of the beta-cell phenotype and maturity onset diabetes.. Genes Dev.

[27] Holland AM, Hale MA, Kagami H, Hammer RE, MacDonald RJ (2002). Experimental control of pancreatic development and maintenance.. Proc Natl Acad Sci U S A.

[28] Prado CL, Pugh-Bernard AE, Elghazi L, Sosa-Pineda B, Sussel L (2004). Ghrelin cells replace insulin-producing {beta} cells in two mouse models of pancreas development.. Proc Natl Acad Sci U S A.

[29] Jacquemin P, Durviaux SM, Jensen J, Godfraind C, Gradwohl G (2000). Transcription factor hepatocyte nuclear factor 6 regulates pancreatic endocrine cell differentiation and controls expression of the proendocrine gene ngn3.. Mol Cell Biol.

[30] Chu K, Tsai MJ (2005). Neuronatin, a downstream target of BETA2/NeuroD1 in the pancreas, is involved in glucose-mediated insulin secretion.. Diabetes.

[31] Arava Y, Adamsky K, Ezerzer C, Ablamunits V, Walker MD (1999). Specific gene expression in pancreatic beta-cells: cloning and characterization of differentially expressed genes.. Diabetes.

[32] Okamoto H (1999). The Reg gene family and Reg proteins: with special attention to the regeneration of pancreatic beta-cells.. J Hepatobiliary Pancreat Surg.

[33] Qiu L, List EO, Kopchick JJ (2005). Differentially expressed proteins in the pancreas of diet-induced diabetic mice.. Mol Cell Proteomics.

[34] De Leon DD, Farzad C, Crutchlow MF, Brestelli J, Tobias J (2006). Identification of transcriptional targets during pancreatic growth after partial pancreatectomy and exendin-4 treatment.. Physiol Genomics.

[35] Goulley J, Dahl U, Baeza N, Mishina Y, Edlund H (2007). BMP4-BMPR1A signaling in beta cells is required for and augments glucose-stimulated insulin secretion.. Cell Metab.

[36] Kassai Y, Munne P, Hotta Y, Penttila E, Kavanagh K (2005). Regulation of mammalian tooth cusp patterning by ectodin.. Science.

[37] Laurikkala J, Kassai Y, Pakkasjarvi L, Thesleff I, Itoh N (2003). Identification of a secreted BMP antagonist, ectodin, integrating BMP, FGF, and SHH signals from the tooth enamel knot.. Dev Biol.

[38] Zhang P, McGrath B, Li S, Frank A, Zambito F (2002). The PERK eukaryotic initiation factor 2 alpha kinase is required for the development of the skeletal system, postnatal growth, and the function and viability of the pancreas.. Mol Cell Biol.

[39] Harding HP, Zeng H, Zhang Y, Jungries R, Chung P (2001). Diabetes mellitus and exocrine pancreatic dysfunction in perk-/- mice reveals a role for translational control in secretory cell survival.. Mol Cell.

[40] Zhang W, Feng D, Li Y, Iida K, McGrath B (2006). PERK EIF2AK3 control of pancreatic beta cell differentiation and proliferation is required for postnatal glucose homeostasis.. Cell Metab.

[41] Teitelman G, Lee J, Reis DJ (1987). Differentiation of prospective mouse pancreatic islet cells during development in vitro and during regeneration.. Dev Biol.

[42] Gu G, Dubauskaite J, Melton DA (2002). Direct evidence for the pancreatic lineage: NGN3+ cells are islet progenitors and are distinct from duct progenitors.. Development.

[43] Gradwohl G, Dierich A, LeMeur M, Guillemot F (2000). neurogenin3 is required for the development of the four endocrine cell lineages of the pancreas.. Proc Natl Acad Sci U S A.

[44] Schwitzgebel VM, Scheel DW, Conners JR, Kalamaras J, Lee JE (2000). Expression of neurogenin3 reveals an islet cell precursor population in the pancreas.. Development.

[45] St-Onge L, Sosa-Pineda B, Chowdhury K, Mansouri A, Gruss P (1997). Pax6 is required for differentiation of glucagon-producing alpha-cells in mouse pancreas.. Nature.

[46] Thomas H, Senkel S, Erdmann S, Arndt T, Turan G (2004). Pattern of genes influenced by conditional expression of the transcription factors HNF6, HNF4alpha and HNF1beta in a pancreatic beta-cell line.. Nucleic Acids Res.

[47] Jacquemin P, Lemaigre FP, Rousseau GG (2003). The Onecut transcription factor HNF-6 (OC-1) is required for timely specification of the pancreas and acts upstream of Pdx-1 in the specification cascade.. Dev Biol.

[48] Moussad EE, Brigstock DR (2000). Connective tissue growth factor: what's in a name?. Mol Genet Metab.

[49] Sommer L, Ma Q, Anderson DJ (1996). neurogenins, a novel family of atonal-related bHLH transcription factors, are putative mammalian neuronal determination genes that reveal progenitor cell heterogeneity in the developing CNS and PNS.. Mol Cell Neurosci.

[50] Pfaff SL, Mendelsohn M, Stewart CL, Edlund T, Jessell TM (1996). Requirement for LIM homeobox gene Isl1 in motor neuron generation reveals a motor neuron-dependent step in interneuron differentiation.. Cell.

[51] Ahlgren U, Pfaff SL, Jessell TM, Edlund T, Edlund H (1997). Independent requirement for ISL1 in formation of pancreatic mesenchyme and islet cells.. Nature.

[52] Osumi N, Hirota A, Ohuchi H, Nakafuku M, Iimura T (1997). Pax-6 is involved in the specification of hindbrain motor neuron subtype.. Development.

[53] Edlund H (1998). Transcribing pancreas.. Diabetes.

[54] Takahashi M, Osumi N (2002). Pax6 regulates specification of ventral neurone subtypes in the hindbrain by establishing progenitor domains.. Development.

[55] Janson J, Laedtke T, Parisi JE, O'Brien P, Petersen RC (2004). Increased risk of type 2 diabetes in Alzheimer disease.. Diabetes.

[56] Grove M, Demyanenko G, Echarri A, Zipfel PA, Quiroz ME (2004). ABI2-deficient mice exhibit defective cell migration, aberrant dendritic spine morphogenesis, and deficits in learning and memory.. Mol Cell Biol.

[57] Giampietro C, Luzzati F, Gambarotta G, Giacobini P, Boda E (2005). Stathmin expression modulates migratory properties of GN-11 neurons in vitro.. Endocrinology.

[58] Gad JM, Keeling SL, Shu T, Richards LJ, Cooper HM (2000). The spatial and temporal expression patterns of netrin receptors, DCC and neogenin, in the developing mouse retina.. Exp Eye Res.

[59] Daniel P, Fitzgerald CSHMC (2006). Localization of Neogenin protein during morphogenesis in the mouse embryo.. Developmental Dynamics.

[60] Tan Y, Yoshida Y, Hughes DE, Costa RH (2006). Increased expression of hepatocyte nuclear factor 6 stimulates hepatocyte proliferation during mouse liver regeneration.. Gastroenterology.

[61] Odom DT, Zizlsperger N, Gordon DB, Bell GW, Rinaldi NJ (2004). Control of pancreas and liver gene expression by HNF transcription factors.. Science.

[62] Rane SG, Dubus P, Mettus RV, Galbreath EJ, Boden G (1999). Loss of Cdk4 expression causes insulin-deficient diabetes and Cdk4 activation results in beta-islet cell hyperplasia.. Nat Genet.

[63] Georgia S, Bhushan A (2004). beta cell replication is the primary mechanism for maintaining postnatal beta cell mass.. J Clin Invest.

[64] Kushner JA, Ciemerych MA, Sicinska E, Wartschow LM, Teta M (2005). Cyclins D2 and D1 are essential for postnatal pancreatic beta-cell growth.. Mol Cell Biol.

[65] Karnik SK, Hughes CM, Gu X, Rozenblatt-Rosen O, McLean GW (2005). Menin regulates pancreatic islet growth by promoting histone methylation and expression of genes encoding p27Kip1 and p18INK4c.. Proc Natl Acad Sci U S A.

[66] Easom RA (2000). Beta-granule transport and exocytosis.. Semin Cell Dev Biol.

[67] Gerber SH, Sudhof TC (2002). Molecular determinants of regulated exocytosis.. Diabetes.

[68] Rhodes CJ, Lucas CA, Mutkoski RL, Orci L, Halban PA (1987). Stimulation by ATP of proinsulin to insulin conversion in isolated rat pancreatic islet secretory granules. Association with the ATP-dependent proton pump.. J Biol Chem.

[69] Gomi H, Mizutani S, Kasai K, Itohara S, Izumi T (2005). Granuphilin molecularly docks insulin granules to the fusion machinery.. J Cell Biol.

[70] Borisovska M, Zhao Y, Tsytsyura Y, Glyvuk N, Takamori S (2005). v-SNAREs control exocytosis of vesicles from priming to fusion.. Embo J.

[71] Martelli AM, Baldini G, Tabellini G, Koticha D, Bareggi R (2000). Rab3A and Rab3D control the total granule number and the fraction of granules docked at the plasma membrane in PC12 cells.. Traffic.

[72] Yi Z, Yokota H, Torii S, Aoki T, Hosaka M (2002). The Rab27a/granuphilin complex regulates the exocytosis of insulin-containing dense-core granules.. Mol Cell Biol.

[73] Kasai K, Ohara-Imaizumi M, Takahashi N, Mizutani S, Zhao S (2005). Rab27a mediates the tight docking of insulin granules onto the plasma membrane during glucose stimulation.. J Clin Invest.

[74] Yaekura K, Julyan R, Wicksteed BL, Hays LB, Alarcon C (2003). Insulin secretory deficiency and glucose intolerance in Rab3A null mice.. J Biol Chem.

[75] Stenmark H, Olkkonen VM (2001). The Rab GTPase family.. Genome Biol.

[76] Varadi A, Tsuboi T, Rutter GA (2005). Myosin Va transports dense core secretory vesicles in pancreatic MIN6 beta-cells.. Mol Biol Cell.

[77] Ching YP, Pang AS, Lam WH, Qi RZ, Wang JH (2002). Identification of a neuronal Cdk5 activator-binding protein as Cdk5 inhibitor.. J Biol Chem.

[78] Ubeda M, Kemp DM, Habener JF (2004). Glucose-induced expression of the cyclin-dependent protein kinase 5 activator p35 involved in Alzheimer's disease regulates insulin gene transcription in pancreatic beta-cells.. Endocrinology.

[79] Wei FY, Nagashima K, Ohshima T, Saheki Y, Lu YF (2005). Cdk5-dependent regulation of glucose-stimulated insulin secretion.. Nat Med.

[80] Irizarry RA, Hobbs B, Collin F, Beazer-Barclay YD, Antonellis KJ (2003). Exploration, normalization, and summaries of high density oligonucleotide array probe level data.. Biostatistics.

