# Supplementary figures and images for: Gene Expression Profiling of a Mouse Model of Pancreatic Islet Dysmorphogenesis
Source: PLoS One. 2008 Feb 20;3(2):e1611. doi: 10.1371/journal.pone.0001611 (PMC2249940; doi:10.1371/journal.pone.0001611)

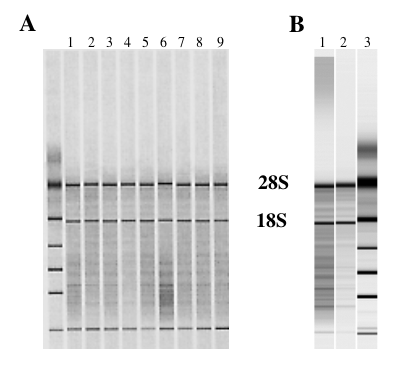

Supplement: Figure S1 — Bioanalysis of individual and pooled RNA samples used for microarray analysis at e18.5. (A) The samples were quantified in 10mM Tris on a Nanodrop ND-1000 Spectrophotometer. In addition to concentration, this instrument reports the 260/280 absorption ratio, which can be an important indicator of hidden contaminants. RNA integrity was assessed on total RNA isolated from individual pancreata at e18.5 using microfluidic analysis. The electropherogram it produces makes it easy to detect certain contaminants or degradation, and to calculate the 28s:18s ratio. Intensity and clarity of 28s and 18s ribosomal RNA bands (indicated) was used as a measure of RNA quality in addition to the lack of degradation products below the 18S band. The far left lane is the molecular weight ladder. Lanes 2, 3, 5, 7, and 8 represent total pancreatic RNA isolated from HNF6 transgenic animals. Lanes 1, 4, 6, and 9 represent total pancreatic RNA isolated from wild type littermates. For microarray analysis, RNA from all transgenic samples were pooled (5 total), while samples 1, 4, and 9 were pooled (3 total) to generate wild type RNA. Sample number 6 was discarded due to the increased presence of degradation products in this sample. (B) Bioanalysis results from the pooled wild type samples (lane 1) and pooled transgenic samples (lane 2). These samples were labeled and used for microarray hybridization. Lane 3 is the molecular weight ladder. (0.46 MB TIF) [file pone.0001611.s001.tif]
